# A Network Meta-Analysis of Clinical Management Strategies for Treatment-Resistant Hypertension: Making Optimal Use of the Evidence

**DOI:** 10.1007/s11606-017-4000-7

**Published:** 2017-03-08

**Authors:** Peter Makai, Joanna IntHout, Jaap Deinum, Kevin Jenniskens, Gert Jan van der Wilt

**Affiliations:** 1 Department for Health Evidence, Health Technology Assessment Group, Radboud Institute for Health Sciences, Radboud University Medical Center, Nijmegen, The Netherlands; 2 Julius Center, University Medical Center Utrecht, Utrecht, The Netherlands

**Keywords:** treatment-resistant hypertension, network meta-analysis, MRAs

## Abstract

**Background:**

With the addition of surgical interventions to current medicinal treatments, it is increasingly challenging for clinicians to rationally choose among the various options for treating patients with apparent treatment-resistant hypertension (ATRHTN).

This study aims to establish the comparative effectiveness of mineralocorticoid receptor antagonists (MRA), renal denervation (RDN), darusentan and central arteriovenous anastomosis (CAA) for patients with ATRHTN by performing a network meta-analysis.

**Methods:**

***Data Sources***: Studies from recent meta-analyses for RDN and placebo effect were supplemented with a systematic search for MRAs in ATRHTN in the Pubmed, EMBASE, CINAHL and Cochrane databases through November 2016.

***Study Selection***: Randomized controlled trials comparing treatment options for patients with ATRHTN.

***Data Extraction and Synthesis***: Data were extracted using predefined data extraction forms, including the Grading of Recommendations Assessment, Development and Evaluation (GRADE) criteria. A Bayesian random effects model was used to conduct a network meta-analysis. Spironolactone was used as the main comparator.

***Main Outcomes and Measures***: Reduction in 24-h ambulatory blood pressure measurement (ABPM).

**Results:**

Twenty articles met our inclusion criteria, and seven treatment alternatives were compared. Compared to MRA, CAA had the highest probability of being more effective, further reducing 24-h SBP (−4.8 mmHg [−13.0, 3.7]) and 24-h DBP (−9.7 mmHg [−18, -0.63]). This difference is likely to be clinically meaningful, with a probability of 78 and 96% at a threshold of a 2-mmHg reduction in blood pressure.

**Conclusions:**

When compared to MRA as anchor, darusentan, CAA and RDN are not more effective in achieving a clinically significant reduction in ambulatory blood pressure in individuals with apparent treatment-resistant hypertension.

**Electronic supplementary material:**

The online version of this article (doi:10.1007/s11606-017-4000-7) contains supplementary material, which is available to authorized users.

## INTRODUCTION

Hypertension (HTN) is the most common condition seen in primary care,^1^ with a global prevalence of 41% in the general population.^2^ Uncontrolled HTN is a risk factor for cardiovascular and renal disease, sources of significant morbidity and cost to society, and is therefore an important public health target.^1^^,^ 3 ^,^ 4 An estimated 14–16% of hypertensive patients have apparent treatment-resistant hypertension (ATRHTN).^5^ This proportion will be even higher if more stringent criteria are adopted for adequate blood pressure control.^6^^–^ 9 ATRHTN is defined as inadequately controlled blood pressure despite receiving three or more adequately dosed hypertensive medications, of which at least one is a diuretic.^10^ Adherence is a key factor in ATRHTN, and according to some estimates, 50% of patients with ATRHTN do not adhere to their medication.^11^^–^ 13 The most common fourth-line treatment is the addition of a mineralocorticoid receptor antagonist (MRA) to the treatment regimen.^14^ In recent years, other pharmacological interventions have been developed, including darusentan,^15^ an endothelin receptor antagonist, as well as various device-based strategies such as renal denervation (RDN) and central arteriovenous anastomosis (CAA).^16^

A lack of data on the comparative effectiveness of these new treatment strategies poses challenges to the production and interpretation of relevant clinical evidence and to choosing optimal treatment strategies for patients. Direct comparison trials and conventional pairwise meta-analyses have demonstrated different conclusions regarding the efficacy of RDN, likely because of the heterogeneity in control treatments. Trials of RDN, for example, have compared RDN with standard medication therapy (no additional treatment),^17^ with a sham procedure (mimicking RDN without actually performing the intervention)^18^ and with MRA as add-on therapy.^19^ While a recent medium-sized trial showed that RDN was superior to MRA,^19^ another trial of the same size failed to show a difference in the primary endpoint.^20^ Among direct comparisons of RDN with other active treatments, results have been mixed. Three conventional pairwise meta-analyses show RDN to be superior,^17^^,^ 21 ^,^ 22 while a fourth meta-analysis, based exclusively on randomized controlled trials (RCTs), casts doubt on these conclusions.^23^

Conventional pairwise meta-analysis is unable to deal with heterogeneity in control interventions. A more appropriate approach in such cases is a network meta-analysis.^24^ In a network-meta analysis, effect sizes associated with direct and indirect comparisons can be estimated for all treatment strategies in the network.^25^ For example, in the case of ATRHTN, if trials have been reported comparing spironolactone (an MRA) with RDN and with darusentan, but no trials have been reported comparing darusentan to RDN directly, an indirect comparison can be made by using the results of the trials in which each was compared with darusentan.^25^ Typically, all comparisons within a network meta-analysis are anchored to a placebo or to a widely accepted active comparator. Using this anchor, it is possible to determine the relative effects of any comparator in the network.

Network meta-analyses can be performed within a comparative effectiveness framework. The main question in this case is not simply whether a new therapy is effective, but whether it is substantially superior to the current standard of care. In the field of ATRHTN, guidelines recommend MRAs,^26^ and therefore we consider this the most suitable anchor within this population.

The aim of this study is to use network meta-analysis to estimate the comparative effectiveness of the third-line hypertensive therapies darusentan, CAA and RDN, compared to a mineralocorticoid receptor antagonist anchor, in reducing systolic and diastolic blood pressure (SBP and DBP, respectively) in patients with ATRHTN.

## METHODS

### Study Identification and Data Extraction

The study was registered in the PROSPERO database, specifying the inclusion criteria and methods used^27^ (PROSPERO 2015:CRD42015017323; available from http://www.crd.york.ac.uk/PROSPERO/display_record.asp?ID=CRD42015017323). To be included in the network meta-analysis, studies were required to meet the following criteria: 1) the study was an RCT; 2) treatment or control treatment were MRA, RDN or placebo (independent of active comparison); 3) the study population comprised patients with treatment-resistant hypertension, defined as uncontrolled hypertension despite receiving three or more antihypertensive medications, of which at least one is a diuretic; 4) the study was published in English; and 5) the study reported 24-h blood pressure obtained by ambulatory blood pressure measurement (ABPM) for SBP and DBP outcomes (absolute or relative to baseline).

Pubmed, EMBASE, CINAHL and Cochrane databases were systematically searched for relevant articles in November 2016. The search terms and strategy are presented in detail in the Online Appendix. PM and KJ independently assessed titles and abstracts, and subsequently selected full-text articles. Records were assessed for eligibility and were excluded in the case of disease condition other than ATRHTN, studies not reporting primary clinical data, or case studies. In addition, two recent systematic reviews on MRAs,^28^^,^ 29 four recent meta-analyses of RDN^17^^,^ 21 ^–^ 23 and one meta-analysis on placebo effect in the ATRHTN population^30^ were checked for relevant references. PM and KJ independently extracted relevant data using a pre-specified form. Main summary measures extracted were study characteristics and average 24-h ABPM. In addition, we extracted data on mean age, sex, study duration and inclusion criteria. Further outcomes extracted were office SBP and DBP (see Online Appendix). For all selected articles, PM and KJ assessed the risk of bias using the Cochrane risk of bias tool^31^ and the Grading of Recommendations Assessment, Development and Evaluation (GRADE) criteria.^32^ All discrepancies between the coders were resolved by consensus.

### Analysis

For the main analysis in the quantitative synthesis, we included studies reporting 24-h ABPM, and excluded studies reporting only office BPM. We have focused on 24-h ABPM, because high-quality evidence was available, and the measurement is more precise and reliable than office BPM. Office BPM was analyzed in a sensitivity analysis. We used a Bayesian random effects network meta-analysis approach to analyze the data, and meta-regression adapted for multiple treatment comparisons. Using meta-regressions, we controlled for the effect of differences in the previously identified variables baseline blood pressure and number of medications used.^30^ Based on the search, the main treatment strategies assigned to the network nodes were: RDN, MRAs (spironolactone and eplerenone), placebo, sham, darusentan, CAA and regimens having more than 3 medications without an add-on. We relate SBP and DBP reduction to the minimum clinically important difference of 2 mmHg.^33^

We used R version 3.3.1^34^ for the analysis, specifically the GeMTC package version 0.8.1^35^ for the meta-analysis.^36^^,^ 37 The network structure was generated using the mtm.networkplot.fun function in R.^38^ We estimated heterogeneity across the network using I^2^. A value of I^2^ below 40% was considered acceptable, while values above 75% were considered as evidence of considerable heterogeneity.^31^ Meta-regression was used to investigate the impact of imbalance in the covariates baseline blood pressure and number of co-medications used, in order to adjust for possible between-study confounders and intransitivity.^24^ We also investigated inconsistency using a node-splitting approach.^39^ Consistent with the use of Bayesian analyses, we report 95% credibility intervals instead of confidence intervals. A 95% credibility interval signifies that there is a 95% probability that the true value lies between the lower and the upper bounds. Thus, our analysis allowed for estimation of the probability that a specific add-on treatment would result in a clinically important lowering of blood pressure in patients with ATRHTN compared to a specified alternative. Details on the estimation procedure are presented in the Online Appendix. Reporting is in accordance with Preferred Reporting Items for Systematic Reviews and Meta-Analyses (PRISMA) guidelines.^40^^,^ 41

## RESULTS

### Study Selection, Characteristics and Risk of Bias

Database searches resulted in 1204 records, and 14 additional records were identified through cross-referencing of previously published meta-analyses (Figure A1). After the removal of duplicates, 984 unique records remained. Twenty studies met the inclusion criteria. Study characteristics are presented in Table 1. The 20 studies allowed comparison of ten medicinal or non-medicinal treatment strategies in the quantitative synthesis. Eplerenone and spironolactone were combined, and a single study with vitamin D was removed from the main analysis due to its ineffectiveness compared with placebo.^42^

**Table 1:** Overview of Included Studies and Their Characteristics, Ordered by Intervention

Study	Inclusion criterion	Method(s) of BP measurement	Intervention	Control	Follow-up (weeks)	Age in years, mean (SD)	Female, *n* (%)	Intervention group (*n*)	Control group (*n*)	BL 24-h SBP, mean (SD) mmHg	BL 24-h DBP, mean (SD) mmHg	BL office SBP, mean (SD) mmHg	BL office DBP, mean (SD) mmHg
Esler 2010^43^	SBP > 160	Office BPM 24-h ABPM	Renal denervation	No additional treatment	24	58 (12)	45 (43%)	52 (20)*	53 (25)*	N/A	N/A	178 (17)	98 (16)
Kario 2015^44^	SBP > 160	Office BPM 24-h ABPM	Renal denervation	No additional treatment	24	58 (6)	10 (24%)	22	19	164 (9)	N/A	180 (13)	93 (12)
Desch 2015^45^	SBP > 135	24-h ABPM	Renal denervation	Sham	24	61 (4)	19 (27%)	35	36	140 (2.6)	79 (2.0)	N/A	N/A
Bhatt 2014^18^	SBP > 160	Office BPM 24-h ABPM	Renal denervation	Sham	24	57 (11)	210 (39%)	364	171	159 (13)	89 (14)	180 (16)	97 (16)
Mathiassen 2016^46^	SBP > 145	Office BPM 24-h ABPM	Renal denervation	Sham	24	56 (9)	18 (26%)	36	33	152 (12)	90 (10)	163 (20)	93 (16)
Azizi 2015^19^	SBP > 140	Office BPM 24-h ABPM	Renal denervation	Spironolactone	24	55 (10)	40 (38%)	48	53	149 (16)	89 (14)	156 (22)	92 (15)
Rosa 2015^20^	SBP > 140	Office BPM 24-h ABPM	Renal denervation	Spironolactone	24	57 (11)	29 (70%)	52	54	148 (12)	85 (10)	157 (18)	90 (14)
Oliveras 2016^47^	SBP > 150	Office BPM 24-h ABPM	Renal denervation	Spironolactone	24	64 (7)	15 (63%)	11	13	153 (9)	81 (9)	156 (9)	84 (10)
Vaclavik 2014^48^	SBP > 140	Office BPM 24-h ABPM	Spironolactone	Placebo	24	60 (10)	52 (35%)	55	56	143 (15)	82 (11)	154 (12)	92 (11)
Oxlund 2013^49^	SBP > 130	Office BPM 24-h ABPM	Spironolactone	Placebo	16	63 (7)	28 (24%)	61	58	144 (10)	78 (7)	144 (15)	78 (10)
Ni 2014^50^	SBP > 140	24-h ABPM	Spironolactone	Placebo	12	55 (13)	31 (41%)	40	36	146 (11)	90 (13)	N/A	N/A
Bobrie 2012^51^	SBP > 135	Office BPM 24-h ABPM	Spironolactone	No additional treatment	12	56 (4)	41 (25%)	85	82	146 (14)	89 (10)	152 (20)	91 (11)
Yang 2016^52^	SBP > 140	Office BPM 24-h ABPM	Spironolactone	No additional treatment	12	44 (13)	N/A	15	15	125 (12)	87 (7)	154 (11)	95 (12)
Black 2007^15^	SBP > 140	Office BPM 24-h ABPM	Darusentan	Placebo	12	62 (10)	47 (41%)	76	39	137 (15)	77 (12)	149 (13)	81 (13)
Bakris 2010^53^	SBP > 140	Office BPM 24-h ABPM	Darusentan	Placebo	14	62 (9)	217 (45%)	364	120	134 (15)	79 (11)	151 (11)	88 (10)
Weber 2009^54^	SBP > 140	Office BPM 24-h ABPM	Darusentan	Placebo	14	62 (9)	191 (52%)	247	132	135 (14)	78 (10)	151 (11)	87 (11)
Karns 2012^55^	SBP > 140	Office BPM 24-h ABPM	Eplerenone	Placebo	8	58 (9)	25 (38%)	33	33	N/A	N/A	154 (9)	90 (11)
Eguchi 2016^56^	SBP > 135	Office BPM 24-h ABPM	Eplerenone	No additional treatment	12	62 (12)	28 37%	40	36	135 (10)	68 (7)	145 (14)	78 (10)
Lobo 2015^16^	SBP > 140	Office BPM 24-h ABPM	Central arteriovenous anastomosis	No additional treatment	24	59 (9)	25 (30%)	44	39	157 (14)	93 (12)	173 (13)	100 (9)

Abbreviations: *BP* blood pressure, *BL* baseline, *SBP* systolic blood pressure, *DBP* diastolic blood pressure, *SD* standard deviation, *BPM* blood pressure measurement, *ABPM* ambulatory blood pressure measurement

* stands for: patients with 24-h blood pressure measurements

Figure A2 shows the risk of bias per study, while Figure A3 shows the risk of bias across studies, according to the GRADE criteria. The studies investigating device-based interventions in particular were not adequately blinded, lacking a sham-controlled study arm.

### Results of Individual Studies

Table 2 shows the results of the individual studies. All treatments significantly reduced blood pressure compared to baseline values. This was also true for the control groups in 8 of the 19 studies, independently of whether an active comparator was used. The between-group difference in 24-h SBP reduction was statistically significant in all studies with the exception of Mathiassen,^46^ Kario,^44^ and SYMPLICITY HTN-3.^18^ This difference was also clinically relevant (blood pressure reduction of 2 mmHg or more) in 13 of the 19 studies. In terms of DBP, there were significant differences in the majority of the studies, the exceptions being the studies by Mathiassen,^46^ Kario,^44^ Desch,^45^ Azizi^19^ and Oxlund.^49^ In 9 of the 19 studies, the between-group differences were clinically relevant.

**Table 2:** Treatment Effects in Studies Reporting 24-h ABP Measurements

Study			Mean (95% CI) change from BL 24-h SBP mmHg	Mean (95% CI) difference between groups 24-h SBP mmHg	Mean (95% CI) change from BL 24-h DBP mmHg	Mean (95% CI) difference between groups 24-h DBP mmHg
Esler 2010^43^	Treatment	Renal denervation	−11 (−15.2, −6.8)	−8.0 (−11.4, −4.6)	−7.0 (−10.1, −3.9)	−6.0 (−8.3, −3.7)
	Control	No additional treatment	−3.0 (−8.2, 2.2)		−1.0 (−4.3, 2.3)	
Kario 2015^44^	Treatment	Renal denervation	−7.5 (−19.5, 4.5)	−6.2 (−13.2, 0.9)	−4.2 (3.2, −11.6)	−3.8 (−8.3, 0.6)
	Control	No additional treatment	−1.4 (−11.6, 8.8)		−0.4 (−7.1, 6.3)	
Bhatt 2014^18^	Treatment	Renal denervation	−6.8 (−8.3, −5.2)	−2.0 (−5.0, 1.0)	−4.1 (−5.1, −3.1)	−1.0 (−1.9, −0.1)
	Control	Sham	−4.8 (−7.4, −2.2)		−3.1 (−4.6, −1.6)	
Desch 2015^45^	Treatment	Renal denervation	−7.0 (−10.8, −3.2)	−3.5 (−4.4, −2.7)	−2.8 (−4.8, −0.9)	−0.7 (−1.2, −0.3)
	Control	Sham	−3.5 (−6.7, −0.2)		−2.1 (−3.9, −0.2)	
Mathiassen 2016^46^	Treatment	Renal denervation	−3.7 (−20.1, 12.7)	1.1 (−5.9, 8.1)	−1.7 (−10.3, 6.9)	0.9 (−2.8, 4.6)
	Control	Sham	−2.6 (−15.4, 10.2)		−2.6 (−10.1, 4.9)	
Rosa 2015^20^	Treatment	Renal denervation	−8.6 (−11.8, −5.3)	−0.5 (−6.1, 5.2)	−5.7 (−7.9, −3.4)	−1.1 (−4.3, 2)
	Control	Spironolactone	−8.1 (−12.7, −3.4)		−4.5 (−6.8, −2.3)	
Azizi 2015^19^	Treatment	Renal denervation	−15.4 (−19.1, −11.7)	−5.9 (−11.0, −0.1)	−9.7 (−12, −7.0)	−3.1 (−6.3, 0.1)
	Control	Spironolactone	−9.5 (−13.0, −6.0)		−6.6 (−8.8, −4.4)	
Oliveras 2016^47^	Treatment	Renal denervation	−5.7 (−14.8, 3.4)	−17.9 (−30.9, −4.9)	−3.7 (−8.2, 0.9)	−6.6 (−12.9, −0.3)
	Control	Spironolactone	−23.6 (−31.9, −15.3)		−10.2 (−14.4 to −6.1)	
Vaclavik 2014^48^	Treatment	Spironolactone	−12.6 (−15.2, 10.0)	−10.5 (−14.6, −6.3)	−5.0 (−7.2, −3.8)	−3.5 (−5.9, −1.0)
	Control	Placebo	−2.1 (−2.3, −1.9)		−2.0 (−3.8, −0.3)	
Oxlund 2013^49^	Treatment	Spironolactone	−9.7 (−13.0, −6.4)	−8.9 (−13.2, −4.6)	−4.2 (−5.8, −2.6)	−3.9 (−6.2, 1.7)
	Control	Placebo	−0.8 (−3.6, 2.1)		−0.3 (−1.9, 1.3)	
Bobrie 2012^51^	Treatment	Spironolactone	−17.0 (−19.2, −14.8)	−10 (−14.0, −6.0)	−10.0 (−11.4, −8.6)	−4.0 (−6, −2)
	Control	No additional treatment	−7.0 (−9.2, −4.8)		−6.0 (−7.3, −4.6)	
Ni 2014^50^	Treatment	Spironolactone	−11.5 (−19.1, −3.9)	−12.5 (−13.8, −11.2)	−7.5 (−14.6, −0.4)	−7.0 (−8.6 to −5.4)
	Control	Placebo	0.5 (−6.4, 7.4)		−1.5 (−9.6, 6.6)	
Yang 2016^52^	Treatment	Spironolactone	−16.3 (−26.3, −6.3)	−11 (−14.2, −7.8)	−14.9 (−23.3, −6.5)	−12 (−14.7, −11.3)
	Control	No additional treatment	−5.3 (−12.3, 1.7)		−2.9 (−9.2, 3.4)	
Karns 2013^55^	Treatment	Eplerenone	N/A	−14.7 (−23.1, −6.3)	N/A	−9.7 (−15.2, −4.2)
	Control	Placebo	N/A		N/A	
Eguchi 2016^56^	Treatment	Eplerenone	−3 (N/A)	−5 (−11.2, 1.2)	−2.5 (N/A)	−4 (−7.4, −0.6)
	Control	No additional treatment	2 (N/A)		1.5 (N/A)	
Black 2007^15^	Treatment	Darusentan	N/A	−9.2 (−11.4, −7.0)	N/A	−7.2 (−8.8, −5.6)
	Control	Placebo	N/A		N/A	
Bakris 2010^53^	Treatment	Darusentan	−9.0 (−21.0, 3.0)	−7.0 (−8.3, −5.7)	−7.5 (−8, −7)	−6.0 (−6.07, −5.9)
	Control	Placebo	−2.0 (−14.0, 10.0)		−1.5 (−2.5, −0.5)	
Weber 2009^54^	Treatment	Darusentan	−17.0 (−20.6, −13.4)	−9.0 (−11.1, −6.9)	−10.0 (−12.1, −7.9)	−5.0 (−6.2, −3.8)
	Control	Placebo	−8.1 (−12.7, −3.4)		−5.0 (−6.7, −3.6)	
Lobo 2015^16^	Treatment	Central arteriovenous anastomosis	−13.5 (−32, 5)	−13.0 (−20.5, −5.5)	−13.5 (−22.0, −6.0)	−13.8 (−22.6, −5.3)
	Control	No additional treatment	−0.5 (−17, 12)		−0.1 (−10.0, 10.0)	

*CI* credibility interval, *BL* baseline

### Synthesis of the Results

**Figure 1:**
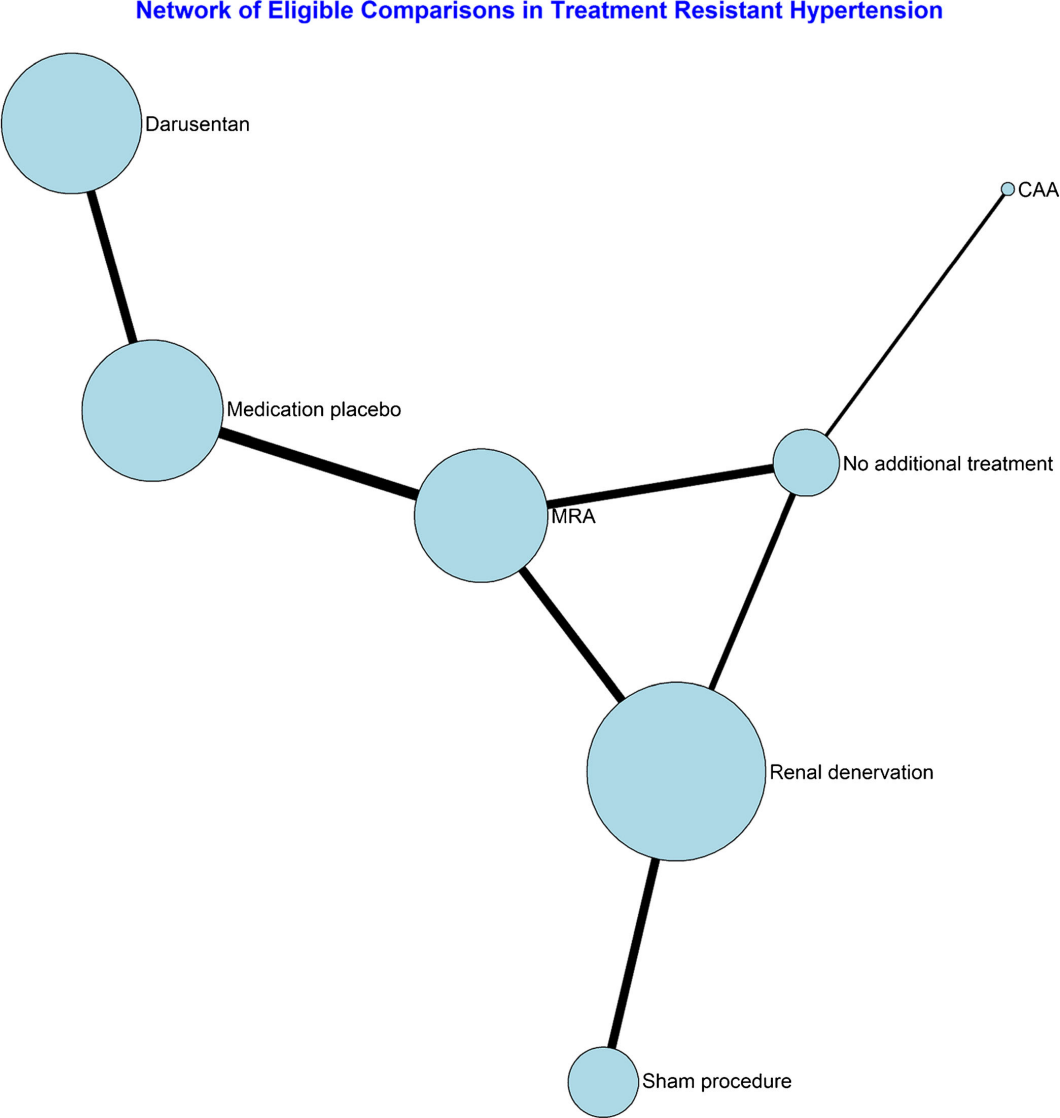
Network structure of studies with available 24-h ABPM.

The network structure is presented in Figure 1. All treatments were add-on therapies, offered to patients with ATRHTN in addition to three or more medications. The size of the circles represents the number of patients per alternative in the network; the lines between the circles show the direct comparisons in the network, with the thickness representing the total number of patients in the trials.

**Figure 2:**
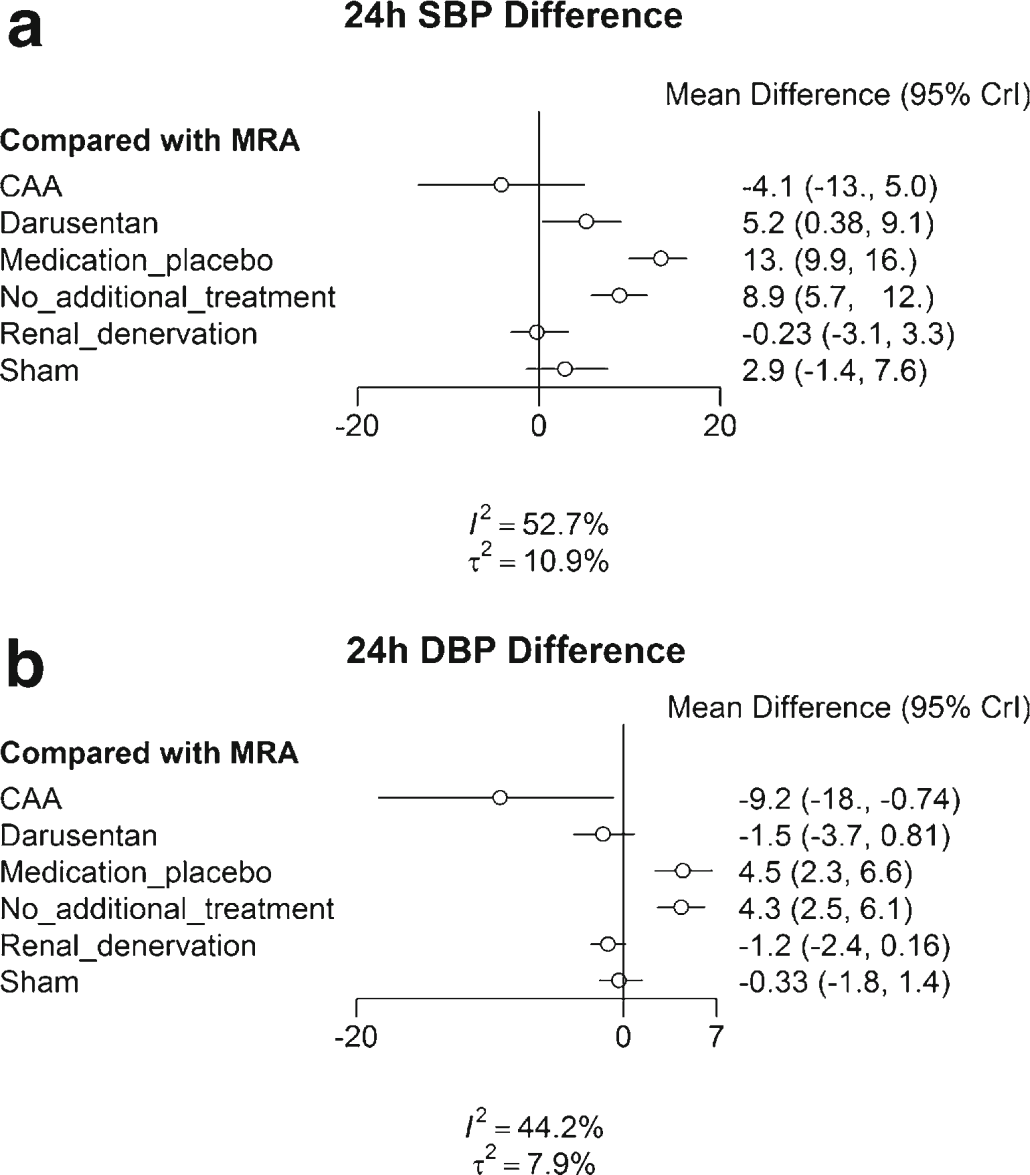
Estimated differences in mean 24-h SBP and DBP (mmHg) reductions, including 95% credibility intervals (CrI). **a** Effectiveness of the various treatment options on the SBP outcome compared to MRA. **b** Effectiveness of the various treatment options on the DBP outcome compared to MRA.

Figure 2 shows the results of the Bayesian random effects network meta-analysis for the 24-h SBP outcome. Since blood pressure reduction is the goal, negative numbers signify higher reduction versus the comparator, and positive values signify a relative increase in blood pressure. Figure 2a and b compares all strategies to spironolactone, with associated 95% credibility intervals (95% CrI).

**Figure 3:**
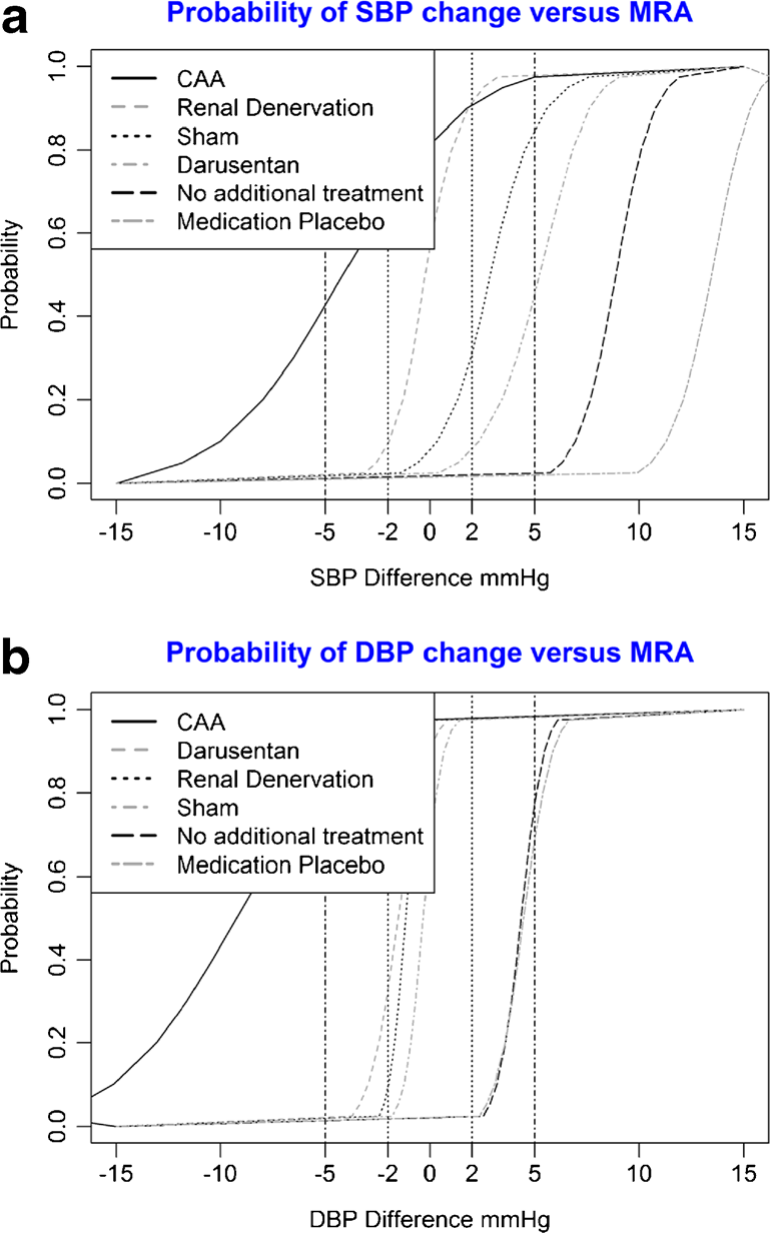
Probability of blood pressure change at various minimum clinically important difference levels. **a** Probability of SBP change versus MRA at various minimum clinically important difference levels. **b** Probability of DBP change versus MRA at various minimum clinically important difference levels.

Figure 3a and b shows the probability of a clinically relevant difference in blood pressure change compared to spironolactone for the 24-h DBP outcome. In Figure 3a and b, the curved lines signify the probability that the difference in blood pressure reduction is larger than the value indicated on the *x*-axis.

#### Effectiveness Compared to MRA

Compared to MRA, darusentan, CAA, and RDN were not more effective in lowering SBP in individuals with treatment-resistant hypertension. (Fig. 2a). The differences in SBP reduction between MRAs, RDN, darusentan and sham procedure were not clinically relevant. MRAs were significantly more effective than medication placebo (−13 mmHg [−9.9, -16.0]) and no additional treatment (−8.9 mmHg [−12.0, -5.7]), and these differences in SBP reduction were also clinically relevant. As shown in Figure 2c, the only treatment that had a reasonable probability of achieving a clinically relevant SBP reduction greater than MRAs was CAA (72%).

For DBP, CAA, Darusentan and RDN achieved significantly greater blood pressure reduction than MRAs (Fig. 2b). Conversely, the difference in DBP reduction due to spironolactone was not clinically relevant when compared with the reduction achieved by darusentan, RDN, and sham procedure. MRAs were significantly more effective than medication placebo −4.4 mmHg [−2.4, -6.5]) and no additional treatment −4.3 mmHg [−2.3, -6.1], and these differences were also clinically relevant. As can be seen on Figure 2d, CAA (92%) had a high probability of achieving a clinically relevant DBP reduction greater than MRAs.

#### Effectiveness Compared to Sham Procedure

Figure A4 shows the comparisons using the sham procedure as an anchor. For SBP, only RDN achieved a significantly greater reduction than the sham procedure. Conversely, the difference in SBP reduction due to the sham procedure was not clinically relevant when compared to darusentan. Sham procedures were more effective than medication placebo and no additional treatment, and these differences were clinically meaningful. As shown in Figure A4C, CAA (90%), RDN (82%) and MRAs (75%) had a reasonable probability of achieving a clinically relevant SBP reduction greater than sham. For DBP, only CAA achieved a significantly greater reduction than the sham procedure. The difference in DBP reduction due to the sham procedure was not clinically relevant when compared to darusentan, RDN and MRAs. Sham procedures were significantly more effective than medication and no additional treatment. As is evident from Figure A4D, CAA (98%) had a reasonable probability of achieving a clinically relevant DBP reduction greater than the sham procedure.

### Risk of Bias Across Studies and Additional Analyses

Heterogeneity in the study results was moderate, with I^2^ = 52.7% for SBP and I^2^ = 44.2% for DBP. The inclusion of baseline covariates such as baseline blood pressure and number of medications used did not significantly impact the results of the meta-analysis, nor could they account for the observed heterogeneity. No evidence of publication bias was found. Office SBP and DBP point estimates were broadly similar to the 24-h ABPM results, with the exception of the CAA and sham procedure results, and the uncertainty around these estimates was much higher. Compared to the use of 24-h SBP/DBP measurements as outcomes, a network meta-analysis of office measurements resulted in a higher level of heterogeneity (I^2^ = 61.2% for OSBP and I^2^ = 99% for ODBP respectively). A sensitivity analysis, discarding studies with more than three indicators of bias, did not produce results that differed from the main analysis.

## DISCUSSION

### Summary of Evidence

#### Comparative Effectiveness

When compared to MRA as an anchor, darusentan, CAA and RDN were not more effective in achieving a clinically significant reduction in ambulatory blood pressure measurement in individuals with ATRHTN. Most individual comparative trials testing a specific medical or non-medical regimen in patients with treatment-resistant hypertension have reported some, albeit variable, benefit in terms of blood pressure control. However, the use of different comparators, including no additional treatment, drug placebo, sham procedure and active comparator, hampers the interpretation of results across studies. Conventional meta-analyses to date have been unable to resolve this problem, because by lumping of control interventions, they ignore the heterogeneity of the comparators.^17^^,^ 21 ^,^ 22 A network meta-analysis can accommodate this type of heterogeneity,^24^ enabling optimal use of the available evidence to guide clinical management. The results of such an approach here show that none of the treatments included in this study resulted in a statistically significant greater SBP reduction compared with MRAs. For DBP, CAA resulted in a clinically relevant and statistically significant greater reduction. The results also allow for an estimate of the probability that either of these treatments result in clinically relevant better blood pressure control. For 24-h SBP, CAA has a 72% probability of resulting in better BP control than MRAs. For 24-h DBP, this figure is 98%. With such figures, individual clinicians and guideline committees can weigh the probability of successful treatment against potential side effects or cost-effectiveness in order to select optimal treatments for patients with treatment-resistant hypertension. An additional advantage of this type of analysis is that such results might be interpreted more easily by patients, facilitating patient–physician communication.

#### Effectiveness Compared to Placebo

Particular care needs to be taken in interpreting the effectiveness of the interventions, as significant and major differences were found between medication placebo and sham procedure, another type of placebo. Compared with medication placebo or no additional treatment, MRAs significantly reduced both SBP and DBP. The magnitude and the significance of the medication placebo adjusted effect was similar to that in a recent study using home blood pressure outcome measurement,^57^ and in line with the effects found in recent meta-analyses.^29^^,^ 58 At the same time, the effects of MRAs on SBP reduction did not differ from the effects of a sham procedure, another kind of placebo. While this may sound counterintuitive, these results are consistent with the literature as far as the difference in effect size between sham procedures and medication placebo both in this population and in general,^30^^,^ 59 ^,^ 60 while both have a marked effect on blood pressure reduction.^30^ It is possible that the enhanced clinical routine^61^ surrounding a sham procedure increases adherence to the background medications in these patients. While it is likely that adherence to medication also improves somewhat under trial conditions, such increased adherence is less likely when an additional medication is introduced to an already busy medication schedule of four chronic medications on average. In any case, it seems appropriate to use a sham procedure as an effectiveness benchmark in ATRHTN for future interventions, as it appears to provide a common benchmark for comparing both medication and device-based treatment strategies.

### Limitations

This review is not comprehensive, as it does not include all possible treatments for ATRHTN, because not all treatment strategies reported 24-h ABPM for the main analysis. We have focused on 24-h ABPM, for which high-quality evidence was available. In the overall GRADE assessment for the 24-h SBP and DBP outcomes, we have not downgraded the studies, and the quality of the evidence remains high. Using GRADE, results for the office SBP/DBP outcome were downgraded mainly due to imprecision and inconsistency (heterogeneity) across studies. This imprecision may be due to the fact that office blood pressure values are not particularly reliable, and are prone to be influenced by white-coat effects.^30^ In addition, inconsistency (heterogeneity) in office SBP and DBP was substantial or considerable,^31^ leading to unreliable conclusions. As a result, treatments reporting only office blood pressure were excluded. Therefore, barostimulation therapy could not be compared in the main analysis, nor could we include the alpha blocker doxazosin or the beta blocker bisoprolol reported in the recent PATHWAY-2 study.^57^ At the same time, we are confident that we have been able to include all relevant studies reporting 24-h ABPM on MRAs, RDN and particularly placebo-controlled studies on any treatment strategy^30^ in the ATRHTN group, and were thus able to make indirect comparisons between these major strategies for the treatment of ATRHTN.

More importantly, we could not determine whether the patients in the individual studies were truly adherent to three or more antihypertensive medications. This is an important limitation, as there is evidence suggesting that only half of all ATRHTN patients fall into the true ATRHTN category, while the other half may have been misdiagnosed because they did not fully adhere to medication.^11^^–^ 13 Furthermore, non-adherence cannot be reliably measured in this population using self-reported questionnaires,^62^ requiring urine tests to monitor adherence.^11^ This may explain why MRAs are not notably more effective than sham. While it is likely that adherence to medication increases during trial conditions, it is also likely that 100% adherence is not obtained, which may lead to an underestimation of the treatment effect of MRA—and all other add-on medication. Consequently, the results may be driven in part by improved adherence rather than the true effect of an additional medication.

Another limitation of this network meta-analysis was the variation in the patient populations among studies, which could have influenced the results. We adjusted for possible sources of heterogeneity between studies^24^ using previously identified variables such as baseline blood pressure and number of co-medications used,^30^ and the results were not sensitive to these variables. However, because of the low number of studies and the association between covariate levels and treatment strategies, we were not able to use multivariable adjustment techniques in the meta-regression.

In addition, there was substantial use of spironolactone in both the treatment and control arms of trials that did not evaluate mineralocorticoid receptor antagonist-based therapies. For example, mineralocorticoid receptor antagonists were used in 22–28% of patients in the SYMPLICITY HTN-3 trial, 17% in the SYMPLICITY HTN-2 trial, and 37–45% of patients in the SYMPLICITY HTN-Japan trial. This contributes to treatment cross-contamination of comparator groups for the indirect pooled comparison of mineralocorticoid receptor antagonists with other groups, and may bias the results towards the null. At the same time, the two moderately large direct comparisons^19^^,^ 20 showed that there was indeed a small difference between the two treatment alternatives, with only the third, very small study (*n* = 24) showing larger differences.^47^

In terms of network consistency, no significant inconsistency was found in the RDN–spironolactone–no additional treatment loop. However, a major limitation of this study is that for most comparisons, there was no combination of direct and indirect evidence: the network contained only a single closed loop enabling comparison of direct and indirect treatments. Therefore, we were only partially able to investigate the consistency of the results; this can be performed fully only if additional trials are reported directly comparing various treatment strategies in the network, thus closing the network’s loops.

## CONCLUSIONS

### Implications for Practice

The only alternative to MRAs which achieved a clinically meaningful blood pressure reduction of 2 mmHg with high probability was CAA. However, the results of CAA are based on a single study, and thus should be interpreted with caution. Also, CAA has frequent adverse effects; therefore, it may not be useful in practice. RDN and darusentan were not clinically different from MRAs.

### Implications for Research

Because inconsistency within the network could only be partially assessed, evidence in the field of ATRHTN would benefit from more direct comparisons of various treatments options, particularly in the case of CAA. In order to improve comparability between strategies, 24-h ABPM should be used in such studies, and adherence should be closely monitored to ensure that only true treatment-resistant hypertension patients are included in the trials.

## Electronic supplementary material

Below is the link to the electronic supplementary material.

ESM 1 (DOCX 206 kb)

## References

[1] James PA, Oparil S, Carter BL, Cushman WC, Dennison-Himmelfarb C, Handler J (2014). 2014 evidence-based guideline for the management of high blood pressure in adults: report from the panel members appointed to the Eighth Joint National Committee (JNC 8). JAMA.

[2] Chow CK, Teo KK, Rangarajan S, Islam S, Gupta R, Avezum A (2013). Prevalence, awareness, treatment, and control of hypertension in rural and urban communities in high-, middle-, and low-income countries. JAMA.

[3] Organization WH. Global health risks: mortality and burden of disease attributable to selected major risks: World Health Organization; 2009

[4] Mancia G, Fagard R, Narkiewicz K, Redon J, Zanchetti A, Böhm M (2013). 2013 ESH/ESC guidelines for the management of arterial hypertension: the Task Force for the Management of Arterial Hypertension of the European Society of Hypertension (ESH) and of the European Society of Cardiology (ESC). Blood Press.

[5] **Achelrod D, Wenzel U, Frey S.** Systematic review and meta-analysis of the prevalence of resistant hypertension in treated hypertensive populations. Am J Hypertens. 2014:hpu151 10.1093/ajh/hpu151 25156625

[6] Wright JT, Williamson JD, Whelton PK, Snyder JK, Sink KM, Rocco MV (2015). A randomized trial of intensive versus standard blood-pressure control. N Engl J Med.

[7] Chobanian AV (2015). Time to Reassess Blood-Pressure Goals. N Engl J Med.

[8] **Bress AP, Tanner RM, Hess R, Colantonio LD, Shimbo D, Muntner P.** Generalizability of results from the Systolic Blood Pressure Intervention Trial (SPRINT) to the US adult population. J Am Coll Cardio. 2015.

[9] Perkovic V, Rodgers A (2015). Redefining blood-pressure targets—SPRINT starts the marathon. N Engl J Med.

[10] Calhoun DA, Jones D, Textor S, Goff DC, Murphy TP, Toto RD (2008). Resistant hypertension: diagnosis, evaluation, and treatment a scientific statement from the American Heart Association Professional Education Committee of the Council for High Blood Pressure Research. Hypertension.

[11] Jung O, Gechter JL, Wunder C, Paulke A, Bartel C, Geiger H (2013). Resistant hypertension? Assessment of adherence by toxicological urine analysis. J Hypertens.

[12] Azizi M, Pereira H, Hamdidouche I, Gosse P, Monge M, Bobrie G (2016). Adherence to Antihypertensive Treatment and the Blood Pressure-Lowering Effects of Renal Denervation in the Renal Denervation for Hypertension (DENERHTN) Trial. Circulation.

[13] Brinker S, Pandey A, Ayers C, Price A, Raheja P, Arbique D (2014). Therapeutic drug monitoring facilitates blood pressure control in resistant hypertension. J Am Coll Cardiol.

[14] Vongpatanasin W (2014). Resistant hypertension: a review of diagnosis and management. JAMA.

[15] Black HR, Bakris GL, Weber MA, Weiss R, Shahawy ME, Marple R (2007). Efficacy and Safety of Darusentan in Patients With Resistant Hypertension: Results From a Randomized, Double‐Blind, Placebo‐Controlled Dose‐Ranging Study. Am J Clin Hypn.

[16] Lobo MD, Sobotka PA, Stanton A, Cockcroft JR, Sulke N, Dolan E (2015). Central arteriovenous anastomosis for the treatment of patients with uncontrolled hypertension (the ROX CONTROL HTN study): a randomised controlled trial. Lancet.

[17] Pancholy SB, Shantha GPS, Patel TM, Sobotka PA, Kandzari DE (2014). Meta-analysis of the effect of renal denervation on blood pressure and pulse pressure in patients with resistant systemic hypertension. Am J Cardiol.

[18] Bhatt DL, Kandzari DE, O’Neill WW, D’Agostino R, Flack JM, Katzen BT (2014). A controlled trial of renal denervation for resistant hypertension. N Engl J Med.

[19] **Azizi M, Sapoval M, Gosse P, Monge M, Bobrie G, Delsart P, et al.** Optimum and stepped care standardised antihypertensive treatment with or without renal denervation for resistant hypertension (DENERHTN): a multicentre, open-label, randomised controlled trial. Lancet. 2015 10.1016/S0140-6736(14)61942-5 25631070

[20] **Rosa J, Widimský P, Toušek P, Petrák O, Čurila K, Waldauf P, et al.** Randomized Comparison of Renal Denervation Versus Intensified Pharmacotherapy Including Spironolactone in True-Resistant Hypertension Six-Month Results From the Prague-15 Study. Hypertension. 2014:HYPERTENSIONAHA. 114.04019 10.1161/HYPERTENSIONAHA.114.04019 25421981

[21] Kwok CS, Loke YK, Pradhan S, Keavney B, El-Omar M, Mamas MA (2014). Renal denervation and blood pressure reduction in resistant hypertension: a systematic review and meta-analysis. Open Heart.

[22] **Zhang X, Wu N, Yan W, Zhou C, Guo H.** The effects of renal denervation on resistant hypertension patients: a meta-analysis. Blood pressure monitoring. 2016 10.1097/MBP.0000000000000177 26901340

[23] **Elmula FEMF, Jin Y, Yang W-Y, Thijs L, Lu Y-C, Larstorp AC, et al.** Meta-analysis of randomized controlled trials of renal denervation in treatment-resistant hypertension. Blood press. 2015(ahead-of-print):1–12 10.3109/08037051.2015.1058595 26194721

[24] Jansen JP, Trikalinos T, Cappelleri JC, Daw J, Andes S, Eldessouki R (2014). Indirect treatment comparison/network meta-analysis study questionnaire to assess relevance and credibility to inform health care decision making: an ISPOR-AMCP-NPC Good Practice Task Force report. Value Health.

[25] Cipriani A, Higgins JP, Geddes JR, Salanti G (2013). Conceptual and technical challenges in network meta-analysis. Ann Intern Med.

[26] Weber MA, Schiffrin EL, White WB, Mann S, Lindholm LH, Kenerson JG (2014). Clinical practice guidelines for the management of hypertension in the community. J Clin Hypertens.

[27] **Makai P, intHout J, Grutters JPC, Deinum J, Jenniskens K, Van der Wilt GJ.** Network meta-analysis of various strategies in treatment resistant hypertension. 2017. http://www.crd.york.ac.uk/PROSPERO/display_record.asp?ID=CRD42015017323 10.1007/s11606-017-4000-7 PMC5515781 28275946

[28] **Liu G, Zheng X, Xu Y, Lu J, Hui R, Huang X.** Effect of aldosterone antagonists on blood pressure in patients with resistant hypertension: a meta-analysis. J Hum Hypertens. 2014 10.1038/jhh.2014.64 25078487

[29] Guo H, Xiao Q (2015). Clinical efficacy of spironolactone for resistant hypertension: a meta analysis from randomized controlled clinical trials. Int J Clin Exp Med.

[30] Patel HC, Hayward C, Ozdemir BA, Rosen SD, Krum H, Lyon AR (2015). Magnitude of Blood Pressure Reduction in the Placebo Arms of Modern Hypertension Trials Implications for Trials of Renal Denervation. Hypertension.

[31] **Higgins JP, Altman DG, Gøtzsche PC, Jüni P, Moher D, Oxman AD, et al.** The Cochrane Collaboration’s tool for assessing risk of bias in randomised trials. BMJ. 2011;343 10.1136/bmj.d5928 PMC3196245 22008217

[32] Guyatt GH, Oxman AD, Vist GE, Kunz R, Falck-Ytter Y, Alonso-Coello P (2008). GRADE: an emerging consensus on rating quality of evidence and strength of recommendations. BMJ.

[33] Whelton PK, He J, Appel LJ, Cutler JA, Havas S, Kotchen TA (2002). Primary prevention of hypertension: clinical and public health advisory from The National High Blood Pressure Education Program. JAMA.

[34] Team RC. R: A language and environment for statistical computing. In: R Foundation for Statistical Computing. Vienna, Austria. 2017. http://www.R-project.org/accessed 01.09.2017

[35] **Gert van Valkenhoef JK.** gemtc. 2017. http://github.com/gertvv/gemtc accessed 01.09.2017

[36] Valkenhoef G, Lu G, Brock B, Hillege H, Ades A, Welton NJ (2012). Automating network meta-analysis. Res Synth Meth.

[37] **DeMaggio C.** Bayesian Analysis for Epidemiologists Part IV: Meta-Analysis. 2017. http://www.columbia.edu/∼cjd11/charles_dimaggio/DIRE/styled-4/styled-11/code-9/accessed 01.09.2017

[38] **Spineli L, Mairgiotis A, Salanti G.** Plot of a treatment comparisons network in R. 2013. http://www.mtm.uoi.gr/index.php/how-to-do-an-mtm/10-how-to-do-an-mtm/15-graphicaldescriptionofanetwork. accessed 01.09.2017

[39] **Valkenhoef G, Dias S, Ades A, Welton NJ.** Automated generation of node-splitting models for assessment of inconsistency in network meta-analysis. Res Synth Meth. 2015 10.1002/jrsm.1167 PMC5057346 26461181

[40] Hutton B, Salanti G, Caldwell DM, Chaimani A, Schmid CH, Cameron C (2015). The PRISMA extension statement for reporting of systematic reviews incorporating network meta-analyses of health care interventions: checklist and explanations. Ann Intern Med.

[41] Moher D, Liberati A, Tetzlaff J, Altman DG (2009). Preferred reporting items for systematic reviews and meta-analyses: the PRISMA statement. Ann Intern Med.

[42] Beveridge LA, Struthers AD, Khan F, Jorde R, Scragg R, Macdonald HM (2015). Effect of vitamin D supplementation on blood pressure: a systematic review and meta-analysis incorporating individual patient data. JAMA Intern Med.

[43] Investigators SH (2010). Renal sympathetic denervation in patients with treatment-resistant hypertension (The Symplicity HTN-2 Trial): a randomised controlled trial. Lancet.

[44] Kario K, Ogawa H, Okumura K, Okura T, Saito S, Ueno T (2015). SYMPLICITY HTN-Japan—First Randomized Controlled Trial of Catheter-Based Renal Denervation in Asian Patients. Circ J.

[45] Desch S, Okon T, Heinemann D, Kulle K, Röhnert K, Sonnabend M (2015). Randomized sham-controlled trial of renal sympathetic denervation in mild resistant hypertension. Hypertension.

[46] **Mathiassen ON, Vase H, Bech JN, Christensen KL, Buus NH, Schroeder AP, et al.** Renal denervation in treatment-resistant essential hypertension. A randomized, SHAM-controlled, double-blinded 24-h blood pressure-based trial. J Hypertens. 2016;34:000 10.1097/HJH.0000000000000977 PMC4933576 27228432

[47] Oliveras A, Armario P, Clarà A, Sans-Atxer L, Vázquez S, Pascual J (2016). Spironolactone versus sympathetic renal denervation to treat true resistant hypertension: results from the DENERVHTA study—a randomized controlled trial. J Hypertens.

[48] Václavík J, Sedlák R, Jarkovský J, Kociánová E, Táborský M (2014). Effect of spironolactone in resistant arterial hypertension: a randomized, double-blind, placebo-controlled trial (ASPIRANT-EXT). Medicine.

[49] Oxlund CS, Henriksen JE, Tarnow L, Schousboe K, Gram J, Jacobsen IA (2013). Low dose spironolactone reduces blood pressure in patients with resistant hypertension and type 2 diabetes mellitus: a double blind randomized clinical trial. J Hypertens.

[50] Ni X, Zhang J, Zhang P, Wu F, Xia M, Ying G (2014). Effects of spironolactone on dialysis patients with refractory hypertension: a randomized controlled study. J Clin Hypertens.

[51] Bobrie G, Frank M, Azizi M, Peyrard S, Boutouyrie P, Chatellier G (2012). Sequential nephron blockade versus sequential renin–angiotensin system blockade in resistant hypertension: a prospective, randomized, open blinded endpoint study. J Hypertens.

[52] Yang L, Zhang H, Cai M, Zou Y, Jiang X, Song L (2016). Effect of spironolactone on patients with resistant hypertension and obstructive sleep apnea. Clin Exp Hypertens.

[53] Bakris GL, Lindholm LH, Black HR, Krum H, Linas S, Linseman JV (2010). Divergent results using clinic and ambulatory blood pressures report of a darusentan-resistant hypertension trial. Hypertension.

[54] Weber MA, Black H, Bakris G, Krum H, Linas S, Weiss R (2009). A selective endothelin-receptor antagonist to reduce blood pressure in patients with treatment-resistant hypertension: a randomised, double-blind, placebo-controlled trial. Lancet.

[55] Karns AD, Bral JM, Hartman D, Peppard T, Schumacher C (2013). Study of Aldosterone Synthase Inhibition as an Add‐On Therapy in Resistant Hypertension. J Clin Hypertens.

[56] **Eguchi K, Kabutoya T, Hoshide S, Ishikawa S, Kario K.** Add-On Use of Eplerenone Is Effective for Lowering Home and Ambulatory Blood Pressure in Drug-Resistant Hypertension. J Clin Hypertens. 2016 10.1111/jch.12860 PMC8031520 27296360

[57] Williams B, MacDonald TM, Morant S, Webb DJ, Sever P, McInnes G (2015). Spironolactone versus placebo, bisoprolol, and doxazosin to determine the optimal treatment for drug-resistant hypertension (PATHWAY-2): a randomised, double-blind, crossover trial. Lancet.

[58] **Dahal K, Kunwar S, Rijal J, Alqatahni F, Panta R, Ishak N, et al.** The Effects of Aldosterone Antagonists in Patients With Resistant Hypertension: A Meta-Analysis of Randomized and Nonrandomized Studies. Am J Hypertens. 2015:hpv031 10.1093/ajh/hpv031 25801902

[59] Kaptchuk TJ, Stason WB, Davis RB, Legedza AR, Schnyer RN, Kerr CE (2006). Sham device v inert pill: randomised controlled trial of two placebo treatments. BMJ.

[60] Kong J, Spaeth R, Cook A, Kirsch I, Claggett B, Vangel M (2013). Are all placebo effects equal? Placebo pills, sham acupuncture, cue conditioning and their association. PLoS ONE.

[61] Finniss DG, Kaptchuk TJ, Miller F, Benedetti F (2010). Biological, clinical, and ethical advances of placebo effects. Lancet.

[62] **Pandey A, Raza F, Velasco A, Brinker S, Ayers C, Das SR, et al.** Comparison of Morisky Medication Adherence Scale with therapeutic drug monitoring in apparent treatment-resistant hypertension. J Am Soc Hypertens. 2015;9(6):420–6.e2 10.1016/j.jash.2015.04.004 26051923

