# Supplementary material for: A Network Meta-Analysis of Clinical Management Strategies for Treatment-Resistant Hypertension: Making Optimal Use of the Evidence
Source: J Gen Intern Med. 2017 Mar 8;32(8):921–30. doi: 10.1007/s11606-017-4000-7 (PMC5515781; doi:10.1007/s11606-017-4000-7)
Supplement: Supplementary file 1 — (DOCX 206 kb) [file 11606_2017_4000_MOESM1_ESM.docx]

# Appendix

## Search strategy

We search the following databases and sources: Pubmed, EMBASE, CINAHL and Cochrane. In addition, we have also searched the grey literature, containing government sources, (FDA, Health Canada, European Medicines Agency) and various clinical trial registrations (WHO clinical trials, clinicaltrials.gov, Current Controlled Trials, and Clinical Study) in order to get a global overview of the trials. In case of studies with unclear reasons for termination, the authors were contacted. We did not include trials which are still recruiting patients.

**Pubmed:**

(“mineralocorticoid receptor antagonist"[all fields] OR “mineralocorticoid receptor antagonists"[all fields] OR ”Mineralocorticoid Receptor Antagonists”[MeSH Terms] OR “aldosterone antagonist"[all fields] OR “aldosterone antagonists"[all fields] OR “Spironolactone"[all fields] OR "Spironolactone"[Mesh] OR “eplerenone"[all fields] OR "eplerenone" [Supplementary Concept] OR “low dose spironolactone”[Title/Abstract]) AND (("randomized controlled trials as topic"[MeSH Terms] OR (“randomized controlled trial”[Publication Type] OR “randomized clinical trial”[Title/Abstract] OR “controlled clinical trial”[Publication Type] OR “randomized”[Title/Abstract] OR “placebo”[Title/Abstract] OR "drug therapy"[Subheading] OR “randomly”[Title/Abstract] OR “trial”[Title/Abstract] OR “groups”[Title/Abstract]) NOT ("animals"[MeSH Terms] NOT “Humans"[MeSH Terms]))) AND ("Medical therapy"[all fields] OR "treatment resistant hypertension"[all fields] OR "Hypertension Resistant to Conventional Therapy" [Supplementary Concept] OR "drug resistant hypertension"[all fields] OR "uncontrolled hypertension"[all fields] OR "resistant hypertension"[all fields] OR "drug refractory hypertension"[all fields] OR “resistant arterial hypertension”[all fields])

## Search results

Figure A1. Search strategy


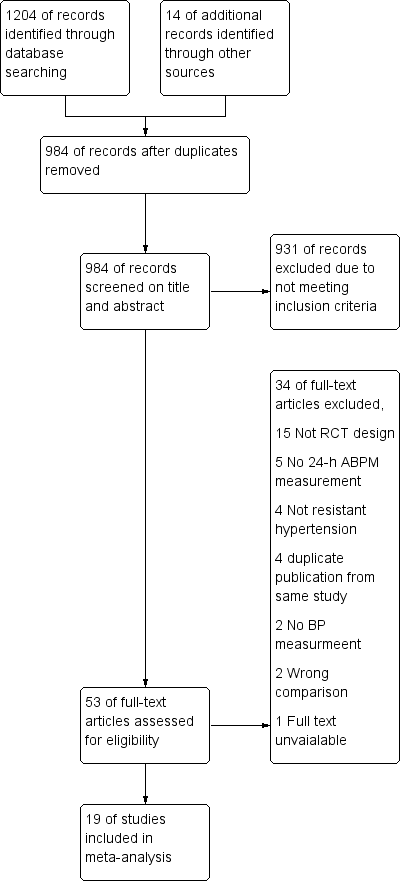


## Risk of bias assessment

Figure A2. Risk of bias per study


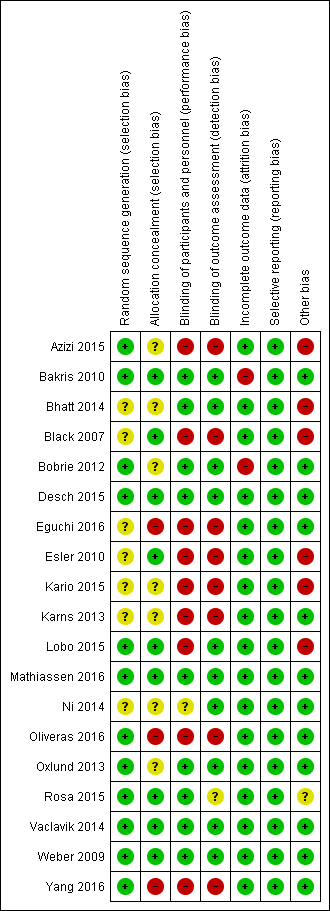


Figure A3 Risk of bias across studies


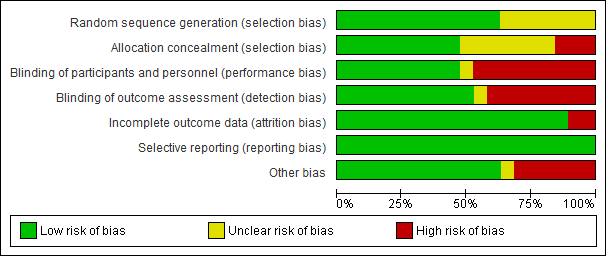


## Statistical analysis

We have performed a Bayesian network meta-analysis. For this purpose, we have used the gemtc version 0.6 package in R version 3.1.2 using JAGS version 3.4, called to R using the rjags package. We set a vague prior for the heterogeneity parameter, specifically we used a gamma prior with a shape parameter of 0.1 and a scale parameter of 0.1. The outcome measure scale to obtain vague priors was set at 50. For the Markov Chain Monte Carlo (MCMC) procedures, we used 50,000 iterations, with 10,000 tuning iterations, and four chains. In addition, we used a thinning factor of 3, keeping every third iteration to limit computer memory use. We checked that the MCMC procedures had reached convergence by visually inspecting the history trace plots, the autocorrelation plots and the cumulative quantile plots for irregularities.

For the Bayesian meta-regression analysis, we used the rjags version 4-6 package in R 3.3.1. rjags calls JAGS version 4.2.0 to R. We specified a Bayesian random-effects meta-regression, with the difference in mean reduction in mm Hg as the outcome variable, the treatments coded as 1 for intervention, 0 if missing from a comparison and as -1 in case a treatment was used as control. In addition, we defined a parameter defining between-study variation. To specify a prior distribution for the mean difference we specified a normal distribution with a mean of 0 and a variance of 900. For the between study precision term (the inverse of the heterogeneity variance) we used a prior uniform distribution between 0 and 0.02. In addition, we specified a prior distribution for each coefficient in the meta-regression, following a normal distribution with a mean 0 and a precision of 0.000001. In order to investigate inconsistency we used an automatic node-splitting approach, as described by Valkenhoef et al. As this is an extension of the previous model, all inputs and priors remain the same.

## Comparisons to sham placebo

Figure A4. Estimated differences in SBP and DBP including 95% Credibility Intervals (CrI) for the mean reductions in 24-h SBP and DBP (mmHg). Effectiveness of the various treatment options when compared to sham (placebo device).

A4A


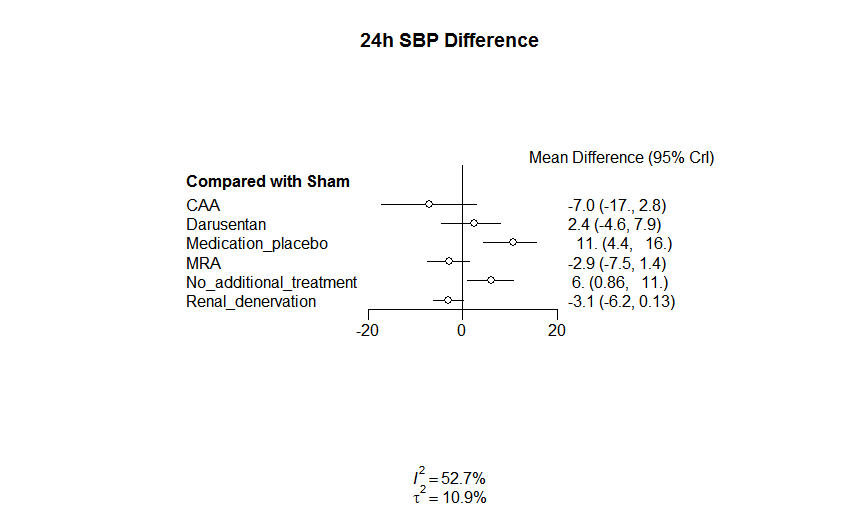


A4B


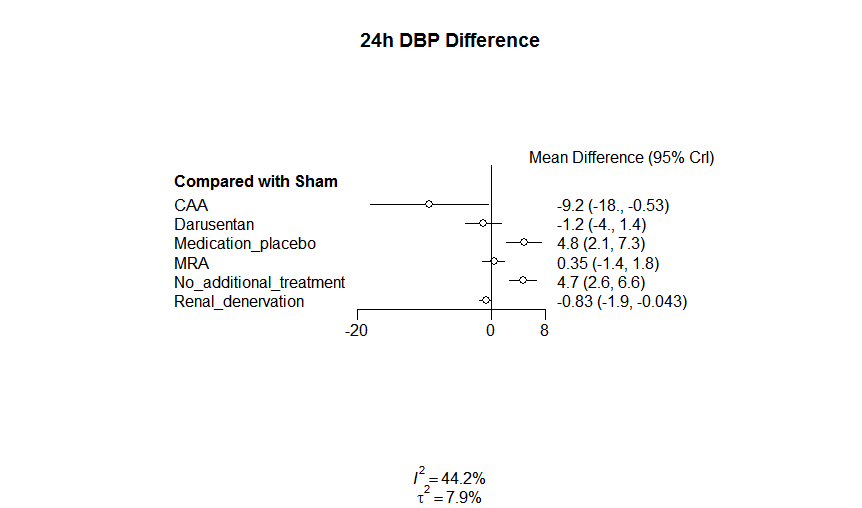


A4C


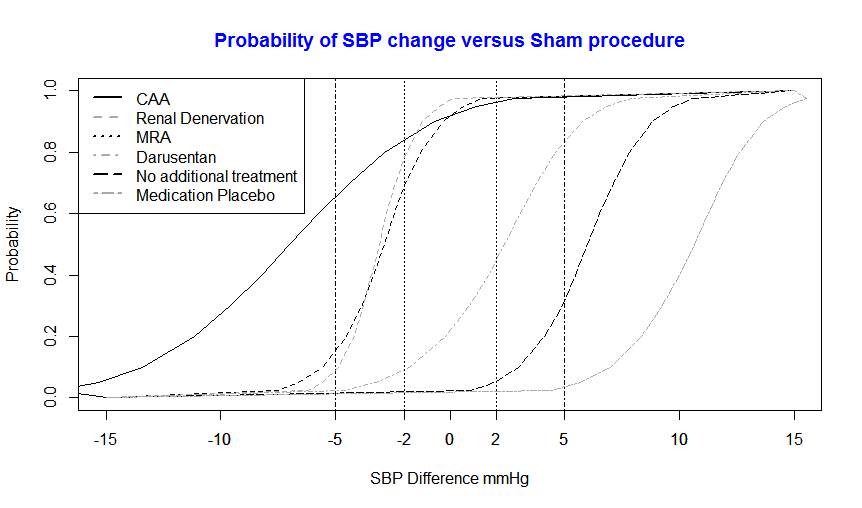


A4D


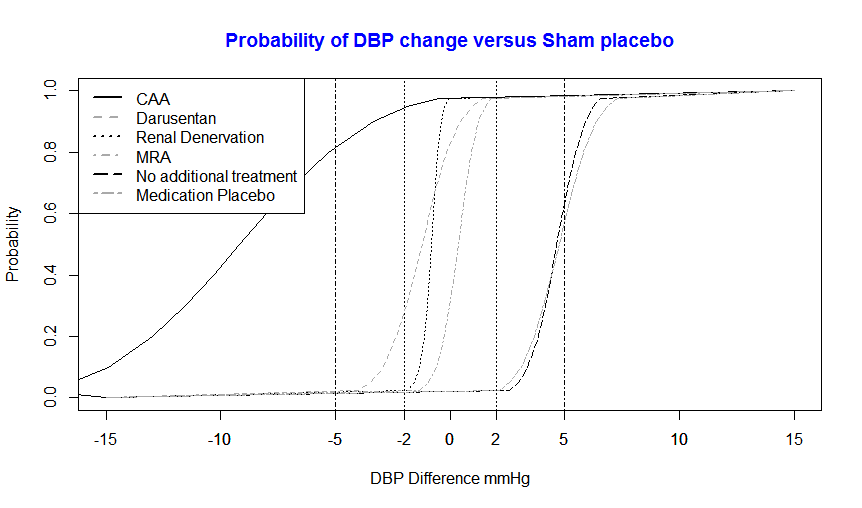


## Office blood pressure measurement

Table A2. Office blood pressure treatments effects of studies included in the network meta-analysis (those reporting 24h ABPM)

| **Study** | **Treatment** | **Control** | **Mean (95% CI)**  **change from BL**  **treatment**  **SBP mmHg** | **Mean (95% CI) change from BL control**  **SBP mmHg** | **Mean (95% CI) difference between groups**  **SBP mmHg** | **Mean (95% CI) change from BL treatment**  **DBP mmHg** | **Mean (95% CI) change from BL control**  **DBP mmHg** | **Mean (95% CI) difference between groups**  **DBP mmHg** |
| --- | --- | --- | --- | --- | --- | --- | --- | --- |
| Esler 2010 | Renal  denervation | No additional treatment | -32 (-38.44, -25.56) | -1 (-6.76, 4.76) | -31 (-41.4, -24.6) | -12.0 (-15.08, -8.92) | 0 (-2.74, 2.74) | -11 (-13.1,-8.9) |
| Kario 2015 | Renal denervation | No additional treatment | -16.6 (-35.1, 1.9) | -7.9(-29.9, 13.1) | -8.64 (-21.12, 3.84) | -5,9 (-17.0, 5.2) | 1 (-7.8, 9.8) | -6.9 (-13.2, 0.5) |
| Bhatt 2014 | Renal  denervation | Sham | -14.13(-16.63, -11.63) | -11.74 (-15.63, -7.85) | -2.39 (-6.89, 2.12) | -6.6 (-7.84, -5.35) | -4.6 (-8.64, -2.56) | -2 (-3.2,-0.8) |
| Desch 2015 | Renal denervation | Sham | n.a. | n.a. | n.a. | n.a. | n.a. | n.a. |
| Mathiassen 2016 | Renal denervation | Sham | n.a. | n.a. | n.a. | n.a. | n.a. | n.a. |
| Azizi 2015 | Renal  denervation | Spironolactone | -15.1 (-20,-9.5) | -9.5 (14.7,-4.2) | -5.6 (-13.3, 2) | -9.1(12.2—6.0) | -6,0(-9--3) | -3.1 (-7.4, 1.2) |
| Rosa 2015 | Renal denervation | Spironolactone | −12.4 (−17.0, −7.8) | −14.3 (−19.7, −8.9) | 1.9 (-5.2, 9) | −7.4 (−11.0, −3.9) | −7.3 (−10.3, −4.2) | -0.2 (-4.8, 4.5) |
| Oliveras 2016 | Renal denervation | Spironolactone | -17.5 (-29.7 to -5.1) | -29.4 (-40.7 to -18.1) | -12.1 (-29.1 to 5.1) | -7.5 (-15.5 to 0.5) | -12.7 (-20.0 to -5.5) | -5.3 (-16.3 to 5.8) |
| ASPIRANT 2014 | Spironolactone | Placebo | -17.6 (-33.1,-2.1) | -7.7(-21.9, 6.7) | -9.9 (-14.6,-5.1) | -7.4 (-8.17, -6.63) | -4.4 (-9.66, -2.35) | -3 (-6.6, 0.7) |
| Oxlund 2013 | Spironolactone | Placebo | -10.5 (-14.5, -6.5) | 5.3 (0.8, 10.0) | -15.8 (-21.8, -9.8) | -5,7(-7,8--3,7) | 1,1(-1,0-3,2) | -6.8 (-9.7, -3.9) |
| Ni 2014 | Spironolactone | Placebo | n.a. | n.a. | n.a. | n.a. | n.a. | n.a. |
| Bobrie 2012 | Spironolactone | No additional treatment | -22 (-24.55, -19.45) | -11 (-13.98, -7.97) | -11 (-15, -6) | -11 (-12.40, -9.60) | -8 (-9.49, -6.48) | -4 (-6,-1) |
| Yang 2016 | Spironolactone | No additional treatment | -27.7 (-41.1, -14.3) | -13.4 (-20.9, -5.9) | -14,3(-18.2, -10.4) | -18.5 (-31.0, -6.0) | -3.4 (-16.3, 9.5) | -15.1(-19.8, -10.4) |
| Black 2007 | Darusentan | Placebo | -17 (-20.60, -11.97) | -6 (-8.78, -2.12) | -11.5 (-14.6,-8.4) | -8.8 (-20.33, -12.36) | -2.5 (-8.78, -2.12) | -6.3 (-8.3,-4.3) |
| Bakris 2010 | Darusentan | Placebo | -15 (-29, -1) | -14 (-28, 0) | -2 (-16, 12) | -10 (-11, -10) | -6 (-6.4, -5.6) | -4 (-4.1, -3.9) |
| Weber 2009 | Darusentan | Placebo | -18 (-22.04, -13.96) | -8 (-10.73, -6.27) | -10 (-14.9 -5.1) | -10 (-12.34, -7.66) | -5 (-6.7, -3.64) | -5 (-6.3, -3.7) |
| Karns 2012 | Eplerenone | Placebo | NA | NA | -9.9 (-13.9 -5.9) | NA | NA | -2.9 (-4.9, 2) |
| Eguchi 2016 | Eplerenone | No additional treatment | -2 (n.a.) | 6 (n.a.) | -8 (-12.5, -3.5) | -2.5 (n.a.) | -2.5 (n.a.) | 0 (-0.8, 0.8) |
| Lobo 2015 | Central arteriovenous anastomosis | No additional treatment | -26.9 (-3, -51) | -3.7 (-25, 18) | -23.2 (-28.9, -17.53) | -20.1 (-34,-5) | -2.4 (-16, 10) | -17.7 (-21.86, -13.21) |

Figure A5. Office SBP


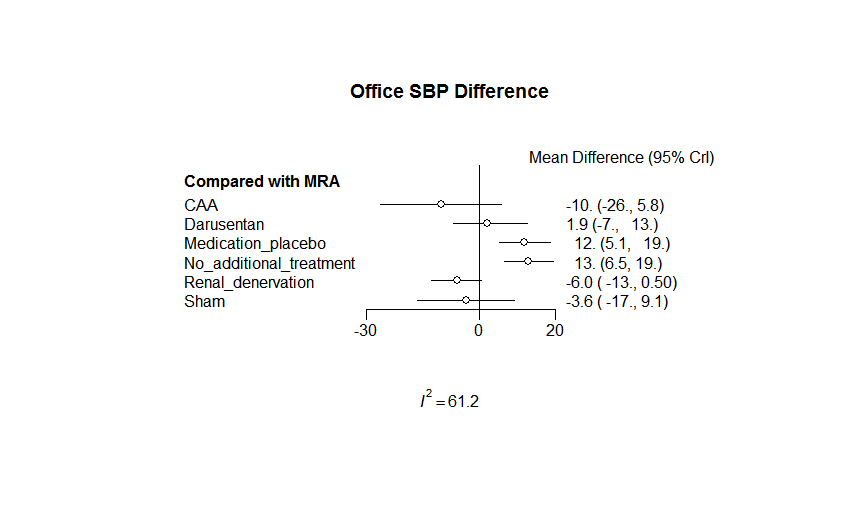


Figure A6. Office DBP


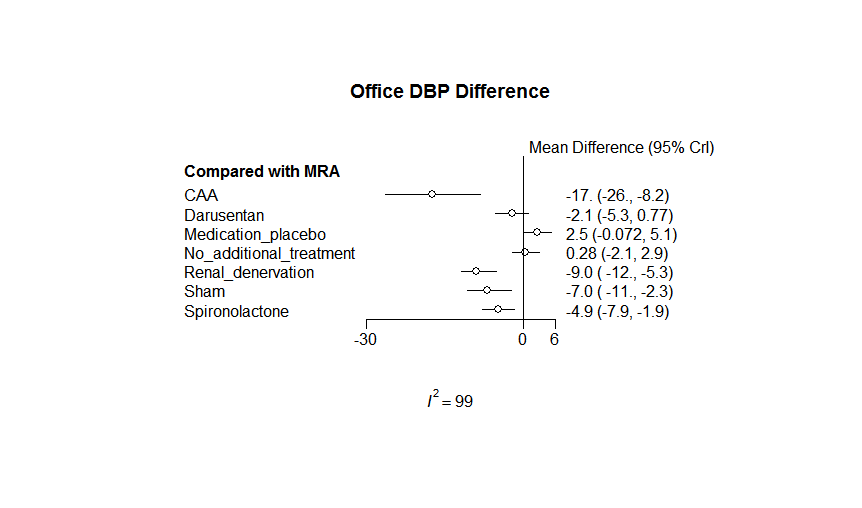


## References Appendix

1. Elmula FEMF, Hoffmann P, Larstorp AC, Fossum E, Brekke M, Kjeldsen SE, et al. Adjusted drug treatment is superior to renal sympathetic denervation in patients with true treatment-resistant hypertension. Hypertension. 2014;63(5):991-9.

2. Investigators SH-**.** Renal sympathetic denervation in patients with treatment-resistant hypertension (The Symplicity HTN-2 Trial): a randomised controlled trial. The Lancet. 2010;376(9756):1903-9.

3. Pokushalov E, Romanov A, Corbucci G, Artyomenko S, Baranova V, Turov A, et al. A randomized comparison of pulmonary vein isolation with versus without concomitant renal artery denervation in patients with refractory symptomatic atrial fibrillation and resistant hypertension. Journal of the American College of Cardiology. 2012;60(13):1163-70.

4. Kario K, Ogawa H, Okumura K, Okura T, Saito S, Ueno T, et al. SYMPLICITY HTN-Japan–First Randomized Controlled Trial of Catheter-Based Renal Denervation in Asian Patients–. Circulation Journal. 2015;79(6):1222-9.

5. Desch S, Okon T, Heinemann D, Kulle K, Röhnert K, Sonnabend M, et al. Randomized sham-controlled trial of renal sympathetic denervation in mild resistant hypertension. Hypertension. 2015;65(6):1202-8.

6. Bhatt DL, Kandzari DE, O'Neill WW, D'Agostino R, Flack JM, Katzen BT, et al. A controlled trial of renal denervation for resistant hypertension. New England Journal of Medicine. 2014;370(15):1393-401.

7. Azizi M, Sapoval M, Gosse P, Monge M, Bobrie G, Delsart P, et al. Optimum and stepped care standardised antihypertensive treatment with or without renal denervation for resistant hypertension (DENERHTN): a multicentre, open-label, randomised controlled trial. The Lancet. 2015.

8. Rosa J, Widimský P, Toušek P, Petrák O, Čurila K, Waldauf P, et al. Randomized Comparison of Renal Denervation Versus Intensified Pharmacotherapy Including Spironolactone in True-Resistant Hypertension Six-Month Results From the Prague-15 Study. Hypertension. 2014:HYPERTENSIONAHA. 114.04019.

9. Václavík J, Sedlák R, Jarkovský J, Kociánová E, Táborský M**.** Effect of spironolactone in resistant arterial hypertension: a randomized, double-blind, placebo-controlled trial (ASPIRANT-EXT). Medicine. 2014;93(27):e162.

10. Abolghasmi R, Taziki O**.** Efficacy of low dose spironolactone in chronic kidney disease with resistant hypertension. Saudi Journal of Kidney Diseases and Transplantation. 2011;22(1):75.

11. Oxlund CS, Henriksen JE, Tarnow L, Schousboe K, Gram J, Jacobsen IA**.** Low dose spironolactone reduces blood pressure in patients with resistant hypertension and type 2 diabetes mellitus: a double blind randomized clinical trial. Journal of hypertension. 2013;31(10):2094-102.

12. Williams B, MacDonald TM, Morant S, Webb DJ, Sever P, McInnes G, et al. Spironolactone versus placebo, bisoprolol, and doxazosin to determine the optimal treatment for drug-resistant hypertension (PATHWAY-2): a randomised, double-blind, crossover trial. The Lancet. 2015;386(10008):2059-68.

13. Bobrie G, Frank M, Azizi M, Peyrard S, Boutouyrie P, Chatellier G, et al. Sequential nephron blockade versus sequential renin–angiotensin system blockade in resistant hypertension: a prospective, randomized, open blinded endpoint study. Journal of hypertension. 2012;30(8):1656-64.

14. Black HR, Bakris GL, Weber MA, Weiss R, Shahawy ME, Marple R, et al. Efficacy and Safety of Darusentan in Patients With Resistant Hypertension: Results From a Randomized, Double‐Blind, Placebo‐Controlled Dose‐Ranging Study. The Journal of Clinical Hypertension. 2007;9(10):760-9.

15. Bakris GL, Lindholm LH, Black HR, Krum H, Linas S, Linseman JV, et al. Divergent results using clinic and ambulatory blood pressures report of a darusentan-resistant hypertension trial. Hypertension. 2010;56(5):824-30.

16. Weber MA, Black H, Bakris G, Krum H, Linas S, Weiss R, et al. A selective endothelin-receptor antagonist to reduce blood pressure in patients with treatment-resistant hypertension: a randomised, double-blind, placebo-controlled trial. The Lancet. 2009;374(9699):1423-31.

17. Karns AD, Bral JM, Hartman D, Peppard T, Schumacher C**.** Study of Aldosterone Synthase Inhibition as an Add‐On Therapy in Resistant Hypertension. The Journal of Clinical Hypertension. 2013;15(3):186-92.

18. Witham MD, Ireland S, Houston JG, Gandy SJ, Waugh S, MacDonald TM, et al. Vitamin D Therapy to Reduce Blood Pressure and Left Ventricular Hypertrophy in Resistant Hypertension Randomized, Controlled Trial. Hypertension. 2014;63(4):706-12.

19. Bisognano JD, Bakris G, Nadim MK, Sanchez L, Kroon AA, Schafer J, et al. Baroreflex activation therapy lowers blood pressure in patients with resistant hypertension: results from the double-blind, randomized, placebo-controlled rheos pivotal trial. Journal of the American College of Cardiology. 2011;58(7):765-73.

20. Lobo MD, Sobotka PA, Stanton A, Cockcroft JR, Sulke N, Dolan E, et al. Central arteriovenous anastomosis for the treatment of patients with uncontrolled hypertension (the ROX CONTROL HTN study): a randomised controlled trial. The Lancet. 2015;385(9978):1634-41.
